# Joint effects of individual reading skills and word properties on Chinese children’s eye movements during sentence reading

**DOI:** 10.1038/s41598-023-41041-4

**Published:** 2023-09-07

**Authors:** Ming Yan, Jinger Pan

**Affiliations:** 1 Department of Psychology, University of Macau, Taipa, Macau China; 2 Center for Cognitive and Brain Sciences, University of Macau, Taipa, Macau China; 3 Department of Psychology, The Education University of Hong Kong, Tai Po, Hong Kong, China

**Keywords:** Human behaviour, Psychology

## Abstract

Word recognition during the reading of continuous text has received much attention. While a large body of research has investigated how linguistic properties of words affect eye movements during reading, it remains to be established how individual differences in reading skills affect momentary cognitive processes during sentence reading among typically developing Chinese readers. The present study set out to test the joint influences of word properties and individual reading skills on eye movements during reading among Chinese children. We recorded eye movements of 30 grade 3 (G3) children and 27 grade 5 (G5) children when they read sentences silently for comprehension. Predictors of linear mixed models included word frequency, visual complexity, and launch site distance, in addition to the participants’ offline psychometric performances in rapid naming, morphological awareness, word segmenting, and character recognition. The results showed that word properties affected word recognition during sentence reading in both G3 and G5 children. Moreover, word segmenting predicted the G3 children’s fixation durations and the G5 children’s fixation location, whereas rapid naming predicted the G5 children’s fixation duration. Implications are discussed based on the current findings, in light of how different literacy skills contribute to reading development.

## Introduction

Text reading is a complex behavior that involves several processes. Word recognition during reading has received much attention in previous research from different perspectives. From a cognitive perspective, researchers are interested mainly in how word-specific linguistic properties affect single-word recognition^1,2^. From a developmental perspective, researchers have identified several specific skills that correlate with word reading, such as phonological awareness, rapid automatized naming (RAN), and morphological awareness^3,4^. However, single-word recognition tasks and psychometric tests may not provide effective reflections of cognitive processes undergone during natural reading in daily life, where texts are written continuously. To this end, the eye-tracking technique provides a powerful tool to reveal the cognitive processes during continuous reading. In this area, a large body of the current research has devoted effort to studying how linguistic properties affect eye movements, based on which relevant computational models have been developed. However, word recognition depends not only on word properties, but also on how well these properties are learned by individual readers^5,6^. Thus, it is not surprising that individual differences in reading skills may affect eye movements during reading. Up to now, only a small number of studies has investigated the effect of individual reading skills on eye movements during reading. These studies either used age as an indicator of reading ability or compared impaired readers with their chronological controls^7,8^. Not many have looked into how word properties and individual reading skills jointly affect eye movements at different stages of reading development, especially in a non-alphabetic script^9,10^. The present study was an attempt to address these issues.

### Linguistic factors on eye movements in reading

During reading of continuously written texts, readers need to send their eyes to different locations of the text to obtain useful information. The eye tracking technique has been proven a sensitive tool to study moment-to-moment cognitive processes during reading. Much psycholinguistic research has focused on the influences of the so-called *big three* linguistic factors, namely word frequency (i.e., how often a word appears in a language), predictability (i.e., how likely a word can be guessed from prior context), and length^11^. In general, readers fixate more briefly on words that are shorter in length, more frequent, and more predictable than longer, less frequent, and less predictable ones^12–15^. As well, word length exercises a strong influence on first-fixation location (FL, where the eye gaze initially lands within a word relative to word beginning) in alphabetic languages^16,17^. Higher levels of word frequency^18,19^ and predictability^20^ (but see Rayner et al.^19^ and Vainio et al.^21^ for null effects), guided readers’ gazes further into the words, leading to FL closer to the word centers. These factors also play a role in children’s eye movements during reading in alphabetic languages^7,10,22,23^. Much of the work on the influences of linguistic factors on oculomotor activities has been conducted using the corpus-analytic approach. In contrast to experimental orthogonal manipulations, corpus analyses estimate a large number of potentially correlated predictors simultaneously. In this case, effects must be controlled statistically for possible influences of the other predictors and individual differences at the levels of readers and items, using statistical models such as linear mixed models^11^.

### Individual reading skills and eye movements

Besides the influence from linguistic properties of the words, research in the field of reading development has indicated a few important predictors (e.g., rapid naming, phonological awareness, morphological awareness) of reading. According to the Verbal Efficiency Theory^5,6^, word recognition is not only affected by properties of individual words, but also how these properties are learned and integrated by an individual reader. Therefore, individual reading skills should be another source of variance observed in eye movements during reading.

Previous studies in alphabetic languages have established that eye movement measures, such as FL, fixation duration and perceptual span (i.e., the area from which useful visual information could be extracted in a single fixation) can differ as a function of reading skills. For instance, Rayner^24^ found that native beginning English readers have a perceptual span of 11 character spaces to the right of a fixation; this is smaller than that of adults, which extends to 14–15 characters to the right of a fixation. Similar patterns that children’s perceptual span increases as they develop have been reported in German^25,26^ and Chinese^27^. In addition, children gradually make shorter fixations as they grow up^28–30^. The same trend was also reported in studies with Chinese children at different developmental stages, who approximate asymptotic levels around G5 and G6^9,23,31–33^.

Other studies have looked at how individual differences in reading skills affect eye movements among readers in the same age groups. Studies of the eye-movement characteristics of dyslexic adults and children have found that they fixate significantly longer on words than their age-matched controls in alphabetic languages when reading sentences^7,34–36^. FLs of dyslexic readers tend to be closer to word beginnings compared to typical readers^35^. Pan and colleagues reported similar findings in a comparison between dyslexic and typically developing Chinese children^37^. Fan and Reilly^9^ also found that individual reading ability affected fixation duration and saccade amplitude among G4 and G5 Chinese children. Besides, previous studies have demonstrated that age-matched dyslexic readers show different patterns of eye movements from their peers, not only in reading but also in the RAN task^38–41^, suggesting delayed lexical access and a reduced perceptual span for dyslexic readers.

There has been only a small number of studies on the relationship between participant-specific reading skills and eye movements among typical readers. Most of these were concerned with alphabetic languages. Reading comprehension, phonological skills, lexical richness, and working memory have been demonstrated to be significant predictors of eye movements^12,42–46^. In recent studies, rapid automatized naming (RAN) has also been found as a significant predictor of eye movement measures, arguably because RAN and continuous reading share many essential features, such as oculomotor planning, saccadic execution, visual and phonological decoding^47^. There were significant correlations between gaze duration and RAN (letters) among German G4 children^10^. RAN and word identification skill were the best predictors of fixation duration and FL for English-speaking non-college-bound adolescents^15^.

### The Chinese orthography and individual reading skills

Characters are the basic units of the Chinese writing system. Irrespective of its visual complexity, each Chinese character occupies the same square shape area. Reading and oculomotor activities across different orthographies largely show similar patterns^48^. During reading of sentences and passages of both logographic and alphabetic scripts, the eye gazes remain relatively still on a word for about 150–300 ms (i.e., a fixation) for information processing before moving to another word. Despite its fundamental difference from alphabetic scripts in the relationships between orthography, phonology, semantics and morphology, linguistic factors also influence lexical access during Chinese sentence reading. For instance, character visual complexity and frequency affected fixation location and duration respectively^9^. In a comparison between dyslexic and typically developing children, word frequency was found to affect fixation duration and word length affected FL^37^. Corpus analyses of eye movements during Chinese sentence reading have also revealed that words’ properties, such as word length, frequency, visual complexity and predictability, are the major predictors of fixation location and duration, just as in other languages^49,50^.

A significant difference between Chinese and alphabetic languages is that Chinese sentences are written without inter-word spaces in text to indicate where a word begins and ends. In spaced scripts such as English, low-level features such as inter-word spaces are the major cues for saccade-target selection towards the word center^51^, an optimal position for lexical processing^52^. Consequently, it is not surprising to see that children as young as G1 can already generate saccades efficiently in a similar pattern to that followed by skilled adult readers in English^30^. Similar findings were later reported in several studies in English as well as in other alphabetic scripts^8,28,53,54^. However, Chinese children demonstrated a late maturation of saccade generation^33^. This is because Chinese readers need to segment a string of continuously written characters into meaningful word units before selecting the word centers and fixating on them^55–57^ (cf. Liu et al.^58^) For instance, Yang et al. reported that Chinese readers acquired more information from a parafoveal character N + 2 (i.e., the second character beyond the currently fixated one) when it belonged to word N + 1 than when it was part of word N + 2^56^. Given that the critical character was in the same eccentric location, their results indicate that parafoveal word segmenting must have been achieved. Differences have been reported in fixation location and duration on a critical string of four Chinese characters with and without word boundary ambiguity^59,60^. These findings suggest that word segmenting is an important skill to master during Chinese reading development, since it can enable Chinese children to send their eye gazes efficiently to word centers for optimal processing. However, such a word segmenting skill may take time to develop. Pan et al. found that G3 children with better word segmenting abilities showed shorter fixation durations, indicating that not all children have fully mastered the skill by G3^61^. They noted that words may not be the primary units during text reading for beginning readers. With increased reading and word segmenting abilities, children gradually shift from character reading to word reading. Only by then do words become the basic units for saccades.

Research on reading development and impairment has identified a few other participant-specific variables, in addition to word segmenting, that significantly predict reading performance. RAN, a task which requires participants to name a list or familiar stimuli (e.g., letter, digits, colors, objects) as accurately and rapidly as possible predicts reading fluency in both deep and shallow alphabetic orthographies as well as Chinese^3,62^, presumably because RAN shares many essential features with reading^47^. For this reason, RAN has recently captured much attention of researchers in the field of eye movements. Faster RAN was associated with shorter gaze duration and rereading time^44^. In addition, as reviewed above, eye movements during the RAN task also differentiate dyslexic readers from typically developing readers in Chinese^40,41^, suggesting the significant role of RAN in Chinese literacy development.

Another important predictor of reading performance in Chinese is morphological awareness. Koda (p.299) defined it as “a learner’s grasp of morphological structure (i.e., the ways in which morphemes are conjoined in words) as well as his or her capability of using this knowledge during morphological processing in visual word recognition”^63^. In modern Chinese, most words are compound words made up of multiple characters^64^. In most cases, one character by itself can correspond to more than one morphemic meaning and thus is morpho-semantically ambiguous. By combining multiple characters into a word, the morphemic meaning of each character is determined. For example, the character 包, when meaning bag, can be used to form words 书包 (school bag), 钱包 (wallet), 背包 (backpack). However, it means ‘to wrap’ in 包裹 (wrap), and ‘to include’ in 包括 (consist of). Thus, timely and correct understanding of the morphemic meanings of characters in words facilitates reading. Morphological awareness in the form of disambiguation thus becomes very important in Chinese reading^4,65,66^. In the present study, we included scores from these psychometric tests as well as reading accuracy, and explored how individual differences jointly influenced oculomotor activities together with linguistic factors.

### The present study

To summarize, word properties and individual reading skills are two sources that affect cognitive and linguistic processes during reading. However, based on existing literature, it is not clear how word properties and different reading skills affect Chinese children’s eye movements during reading, and whether they impose different weightings at different stages of their reading development. The present study set out to investigate this question by testing different reading skills among G3 and G5 Chinese children. We chose G3 because previous studies have shown a transition from ‘learning to read’ to ‘reading to learn’ at this grade in alphabetic scripts^67^ as well as in Chinese^68^. G5 was chosen because children at this age usually have similar eye-movement patterns to those of adults, suggesting that they are relatively mature in oculomotor control^23,32^. We tested the influence on these children’s eye movements of two important word-specific linguistic factors, namely, word frequency and visual complexity (i.e., number of strokes), as well as four important reading skills (i.e., word segmenting, RAN, morphological awareness, and character recognition). Given previous research, we predicted that both word properties and individual reading skills would affect readers’ eye movement characteristics. The influence of word properties would be similar across grades: We predicted that fixation duration would decrease, and the eyes would land further, for frequent words and for visually simple words. The effects on individual reading skills on different eye movement measures may vary across the two groups of children. This is because G3 and G5 readers are at different stages of reading development, during which different skills might be needed.

## Methods

### Participants

Fifty-seven children, including 30 G3 children (*M*_age_ = 102.8 months, *SD* = 4.12 months) and 27 G5 (*M*_age_ = 128.4 months, *SD* = 4.96 months) children in a primary school in Beijing, were recruited. All participants were native speakers of Chinese, with normal or corrected-to-normal vision and without any known reading-related deficits. The study was approved by the Institutional Review Board of the State Key Laboratory of Cognitive Neuroscience and Learning in the People’s Republic of China. Written informed consent was obtained from the parents of the children participants prior to the experiment. All experiments were performed in accordance with relevant guidelines and regulations.

### Materials

#### Morphological awareness

The morphological production task was used to measure the children’s morphological awareness^65^. A target character embedded in a two-character word was presented orally to the children. They were asked to produce two different words that contained the target character. In one word, the target character should share the same morpheme that was in the previously-presented word. In the other word they were asked to produce, the target character should be of a different morpheme. For example, a target character 面 in an orally presented word 面包 (bread) means flour. 面粉 (flour) and 面容 (face) are two possible answers for same and different morphemes, respectively. There were 15 target characters and each correct answer was given one point. The maximum score for this task was 30.

#### Character recognition

There were 150 characters ordered in terms of difficulty. It was expected that all of them would have been taught by G6^65^. The children were asked to read out the characters one by one. The test stopped when 15 consecutive characters were named incorrectly. One point was awarded for each correctly named character, and the maximum score was 150.

#### Rapid automatized naming (RAN)

Five numbers (i.e., 1, 2, 3, 5, and 8) were used as stimuli. These numbers were repeated randomly ten times. The children were asked to name the numbers as accurately and rapidly as possible. They named the list twice and the average of the two naming times was used as the score for this task.

#### Word segmenting

Word segmenting ability was assessed using the Word Chain test^69^. A list of character strings was presented to the participants. Each string consisted of three Chinese words, varying in length. They were asked to segment the character strings into words by putting slashes between words as quickly as possible within the given time [for instance, 身体熊蛋白质should be segmented as 身体(body)/熊(bear)/蛋白质(protein)]. The G3 children were given 70 s and the G5 children were given 60 s. We calculated the number of words correctly segmented in one minute. The scores were used to indicate the child’s word segmenting ability.

#### Sentence reading with eye movements recorded

Each participant read 120 sentences while their eye movements were recorded. These sentences were 13–18 characters in length (*M* = 14.7, *SD* = 1.3) and the distribution of word length, which varied from one to four characters, was representative of the Chinese writing system. The numbers of strokes per word, which reflect visual complexity, ranged from 1 to 44 (*M* = 13.5, *SD* = 6.2). Because more than 70% of words were two characters long, we focused on two-character words in our analyses. The eye-movement data were reported previously by Yan et al.^33^, who compared G3, G5 and adult readers to document the developmental trend of saccade-target selection in reading Chinese. In this study, we focused on how linguistic properties and individual reading skills jointly influenced the Chinese children’s lexical processing.

### Apparatus

In the eye tracking experiment, an EyeLink 1000 desktop system (1000 Hz sample rate) was used to record the participants’ eye movements. The participants were seated with their heads positioned on a forehead-and-chin rest in front of a 21-in. CRT monitor (resolution: 1024 by 768 pixels; frame rate: 100 Hz). The distance between the participant’s eyes and the display was 80 cm. Each trial contained one single sentence occupying only one horizontal line on the screen. The font Song was used and each character occupied approximately 1.1 degrees of visual angle. Calibrations and recordings were done monocularly (right eye) and viewing was binocular.

### Procedure

The participants were tested individually, both in the sentence-reading session and the psychometric-measure session. In the sentence-reading session, the participants read 60 sentences in each of two separate blocks with a 30-min break between the two blocks. Each participant received a different random order of sentence presentation. The participants were calibrated with a standard nine-point grid. After validation of calibration accuracy, a fixation-point appeared on the left side of the monitor, where the first character of the sentence would appear if the eye tracker identified the reader’s gaze on the fixation point. The participants were instructed to read the sentence for comprehension, then to fixate at a point in the lower right corner of the screen, and to press a joystick button to indicate the completion of reading a sentence. For 32 (27%) of the sentences, there was a simple yes or no follow-up question to encourage the participants’ engagement with the reading task. The participants needed to press one of the two buttons to answer the questions.

### Data analysis

One G5 child did not complete the individual reading skills measures. His/her data were not included in the analyses. Thus, data of 26 G5 children were analyzed.

For eye-movement data, an algorithm for binocular saccade detection^70^ was used to determine fixations. Following standard procedures, we excluded from the analyses sentences containing missing samples, participants’ blinks, or body movements during the data collection (*N* = 246, 3.7% of all sentences). Sentences with less than 1/3 of the words being fixated on were removed (*N* = 36, 0.06% of all sentences). The first and the last words and the first and the last fixated words in the sentences were not analyzed (*N* = 526, 3.9%). We also excluded words with first-fixation duration (FFD, the duration of the first fixation on a word, irrespective of the number of fixations in first-pass reading) shorter than 60 ms or longer than 800 ms or gaze duration (GD, accumulative durations of fixations during first-pass reading) longer than 1200 ms (*N* = 2741, 6.1%). In addition, we excluded observations with a launch-site distance (i.e., the difference between the last fixation location and the beginning of the currently fixated word) of more than four characters (*N* = 3647, 6.9%), as these observations may reflect oculomotor or tracker errors. Overall, 21,638 observations (11,173 observations for G3 and 10,465 for G5, respectively) were analyzed. The dependent variables were FFD, single-fixation duration (SFD, fixation duration of a word when it receives only one fixation in first-pass reading), GD, and FL.

Linear mixed models (LMMs) from the lmer program of the lme4 package^71^ (Version 1.1-27.1) were used to analyze the data. All analyses were conducted in the R environment for statistical computing and graphics^72^ (Version 4.1.0). Fixed effects included grade specified with a sum contrast, word frequency (log10 transformed), number of strokes, character recognition, morphological awareness, and rapid naming. We also included launch-site distance as a fixed effect, as it has been shown to influence fixation duration and location in previous research^73^. All continuous predictors were centered to their mean values. Subject- and item-related variance components for intercepts and random slopes for fixed effects were included as random effects. We started with models with all random effects and correlation parameters. Parameters with small variances were removed for successful model convergence^74^. *P*-values were obtained using the lmerTest package^75^ (Version 3.1-0) in the R statistical environment. Following previous research, we used log-transformed fixation duration dependent variables in the models^76^.

## Results

The means and standard deviations of different measures are shown in Table 1 for G3 and G5, respectively. As can be seen from Table 1, the children named more characters correctly, had better morphological awareness, needed less time to finish the RAN task and were able to segment more character strings in the word segmenting task from G3 to G5 (*p*s < 0.001).

**Table 1:** Means, standard deviations of different measures.

	G3		G5	
	*M*	*SD*	*M*	*SD*
CR	91.07	17.89	116.54	13.76
MA	18.4	3.07	21.0	3.61
RAN	22.00	4.00	16.74	3.43
WS	9.69	3.77	17.15	4.99
FFD	293	36	253	27
SFD	299	41	253	29
GD	474	52	349	52
FL	.70	.09	.81	.08

*CR* character recognition, *MA* morphological awareness, *RAN* rapid automatized naming (s), *WS* word segmenting (no. of correct answers), *FFD* first-fixation duration (ms), *GD* gaze duration (ms), *TRT* total reading time (ms), *FL* first-fixation location (characters).

Table 2 shows the main effects and interactions. G5 children fixated shorter on and landed further into words as compared to G3 children. Replicating previous findings, the main effects of launch-site distance, word frequency, and number of strokes were significant for all dependent variables, except that the effect of launch-site distance on SFD was not significant. The main effect of word segmenting on GD was significant, suggesting that children who could segment words better fixated shorter on words. The main effects of RAN on FFD and character recognition on FL were marginally significant, suggesting that children with better reading skills had shorter fixation durations and landed further into words. No other main effects of reading ability were significant.

**Table 2:** Outputs of models.

	First-fixation duration				Single fixation duration				Gaze duration				First-fixation location			
	*b*	*SE*	*t*	*p*	*b*	*SE*	*t*	*p*	*b*	*SE*	*t*	*p*	*b*	*SE*	*t*	*p*
Intercept	5.558	0.024	236.333	< 0.001	5.557	0.029	190.050	< 0.001	5.897	0.026	230.840	< 0.001	0.779	0.032	24.648	< 0.001
Grd	− 0.097	0.039	− 2.493	0.016	− 0.108	0.044	− 2.448	0.018	− 0.205	0.047	− 4.390	< 0.001	0.107	0.042	2.554	0.013
LS	− 0.011	0.005	− 2.104	0.040	− 0.007	0.007	− 1.046	0.301	0.037	0.010	3.703	< 0.001	− 0.238	0.012	− 20.188	< 0.001
Freq	− 0.027	0.003	− 7.844	< 0.001	− 0.027	0.004	− 6.034	< 0.001	− 0.051	0.005	− 10.689	< 0.001	0.018	0.004	4.046	< 0.001
Strk	0.002	0.001	4.164	< 0.001	0.005	0.001	6.211	< 0.001	0.012	0.001	14.612	< 0.001	− 0.008	0.001	− 11.238	< 0.001
CR	0.033	0.024	1.399	0.167	0.035	0.027	1.297	0.200	− 0.002	0.003	− 0.071	0.944	0.043	0.026	1.681	0.098
MA	0.009	0.018	0.508	0.614	0.004	0.020	0.208	0.836	− 0.008	0.021	− 0.394	0.695	0.005	0.019	0.255	0.800
RAN	0.040	0.020	1.978	0.053	0.037	0.023	1.591	0.118	0.015	0.024	0.597	0.553	0.034	0.022	1.553	0.126
WS	− 0.030	0.022	− 1.368	0.177	− 0.034	0.025	− 1.372	0.176	− 0.057	0.026	− 2.177	0.034	0.026	0.024	1.096	0.278
Grd:LS	0.003	0.011	0.253	0.801	0.000	0.014	0.008	0.994	0.022	0.020	1.102	0.276	− 0.031	0.024	− 1.314	0.194
Grd:Freq	0.003	0.006	0.436	0.665	0.001	0.008	0.159	0.874	− 0.004	0.009	− 0.507	0.612	− 0.001	0.008	− 0.179	0.858
Grd:Strk	0.003	0.001	2.705	0.007	0.001	0.001	0.679	0.497	− 0.001	0.002	− 0.583	0.560	0.001	0.001	0.872	0.383
Grd:CR	− 0.007	0.048	− 0.149	0.882	0.001	0.053	0.020	0.984	0.024	0.057	0.417	0.679	− 0.032	0.051	− 0.618	0.539
Grd:MA	− 0.054	0.036	− 1.509	0.137	− 0.056	0.040	− 1.402	0.167	− 0.023	0.043	− 0.527	0.600	− 0.016	0.038	− 0.408	0.685
Grd:RAN	0.070	0.041	1.713	0.092	0.067	0.046	1.466	0.148	− 0.000	0.049	− 0.001	0.999	0.035	0.044	0.788	0.434
Grd:WS	0.085	0.044	1.956	0.056	0.104	0.049	2.115	0.039	− 0.033	0.052	− 0.631	0.530	0.103	0.047	2.183	0.033

*Grd* grade, *LS* launch site distance, *Freq* word frequency, *Strk* No. of strokes, *CR* character recognition, *MA* morphological awareness, *RAN* rapid automatized naming (s), *WS* word segmenting (no. of correct answers).

The interactions between grade and number of strokes, grade and RAN, and grade and word segmenting in FFD, between grade and word segmenting in SFD and FL were (marginally) significant. Table 3 shows the simple effects for G3 and G5 children respectively. In both grades, launch-site distance was the primary source of variance in FL. The closer the eyes were to the words, the further they landed into these words. This also predicted the GDs of the G5 children. The further away their eyes were from the words, the longer they fixated on them. Word frequency predicted all fixation duration and location measures in both G3 and G5. Higher frequency words were fixated on with shorter durations and further FLs. Visual complexity also predicted most of the dependent variables in both groups, except FFD among the G3 children. Words that were visually more complex were fixated on longer, and FLs were closer to word beginnings than words that were simple in their visual forms. In general, the effects of word properties on fixation duration and location were similar and these effects replicated robust findings from previous research.

**Table 3:** Simple effects for G3 and G5 children, respectively.

	First-fixation duration				Single fixation duration				Gaze duration				First-fixation location			
	*b*	*SE*	*t*	*p*	*b*	*SE*	*t*	*p*	*b*	*SE*	*t*	*p*	*b*	*SE*	*t*	*p*
G3																
Intercept	5.608	0.022	259.780	< 0.001	5.620	0.025	225.486	< 0.001	6.023	0.023	266.968	< 0.001	0.736	0.033	22.532	< 0.001
LS	− 0.011	0.007	− 1.533	0.137	− 0.006	0.011	− 0.548	0.589	0.028	0.017	1.678	0.104	− 0.222	0.016	− 14.129	< 0.001
Freq	− 0.027	0.005	− 4.932	< 0.001	− 0.025	0.007	− 3.681	< 0.001	− 0.045	0.007	− 6.653	< 0.001	0.017	0.006	2.978	0.003
Strk	0.001	0.001	1.090	0.276	0.004	0.001	3.462	< 0.001	0.012	0.001	10.530	< 0.001	− 0.009	0.001	− 8.913	< 0.001
CR	0.033	0.026	1.296	0.205	0.043	0.024	1.783	0.086	− 0.010	0.027	− 0.370	0.714	0.051	0.026	1.938	0.062
MA	0.030	0.023	1.308	0.201	0.014	0.022	0.608	0.548	0.000	0.024	0.004	0.997	0.009	0.024	0.361	0.720
RAN	0.005	0.022	0.233	0.818	0.021	0.020	1.061	0.298	0.018	0.022	0.794	0.433	0.016	0.022	0.726	0.473
WS	− 0.048	0.022	− 2.202	0.036	− 0.066	0.021	− 3.134	0.004	− 0.031	0.023	− 1.369	0.181	− 0.013	0.023	− 0.576	0.569
G5																
Intercept	5.492	0.023	236.303	< 0.001	5.503	0.029	189.228	< 0.001	5.753	0.029	196.076	< 0.001	0.831	0.028	29.888	< 0.001
LS	− 0.011	0.008	− 1.370	0.183	− 0.008	0.009	− 0.882	0.386	0.048	0.011	4.427	< 0.001	− 0.252	0.018	− 14.142	< 0.001
Freq	− 0.025	0.004	− 5.770	< 0.001	− 0.027	0.005	− 4.896	< 0.001	− 0.056	0.006	− 8.802	< 0.001	0.017	0.006	2.618	0.009
Strk	0.004	0.001	4.859	< 0.001	0.005	0.001	5.204	< 0.001	0.011	0.001	9.936	< 0.001	− 0.008	0.001	− 7.140	< 0.001
CR	0.019	0.022	0.841	0.408	0.024	0.025	0.966	0.343	0.008	0.033	0.241	0.811	0.018	0.027	0.677	0.504
MA	− 0.020	0.022	− 0.861	0.397	− 0.025	0.025	− 0.999	0.327	− 0.020	0.033	− 0.626	0.537	− 0.004	0.027	− 0.150	0.882
RAN	0.054	0.022	2.512	0.019	0.054	0.024	2.264	0.032	0.011	0.031	0.362	0.721	0.037	0.026	1.459	0.157
WS	0.011	0.023	0.484	0.633	− 0.017	0.026	0.652	0.524	− 0.066	0.034	− 1.920	0.066	0.067	0.028	2.389	0.025

*LS* launch site distance, *Freq* word frequency, *Strk* No. of strokes, *CR* character recognition, *MA* morphological awareness, *RAN* rapid automatized naming (s), *WS* word segmenting (no. of correct answers).

**Figure 1:**
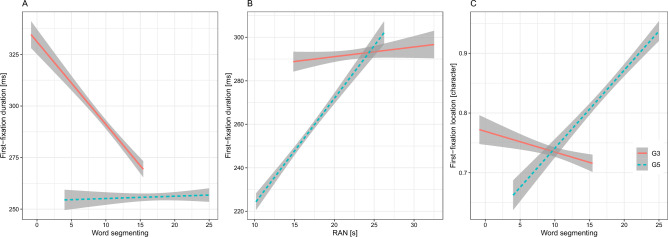
Left panel (**A**): Word segmenting as a predictor of first-fixation duration; Middle panel (**B**): Rapid naming as a predictor of first-fixation duration; Right panel (**C**): Word segmenting as a predictor of first-fixation location.

For individual reading skills among the G3 readers, we found that word segmenting was important for FFD and SFD (see Fig. 1A). Children that were better at segmenting words had shorter fixation durations than those who performed poorly at this task. For the G5 children, RAN predicted FFD and SFD. Children who named the digits faster in the RAN task showed shorter fixation durations than those who were slow in the task (see Fig. 1B). As can be seen in Fig. 1C, word segmenting predicted FL among the G5 children, suggesting that children with better word segment abilities were able to program saccades further into the words than those who were lagging behind in this task. Neither character recognition nor morphological awareness significantly predicted any dependent variables.

## Discussion

Reading is a complex task. Recognizing words during the reading of continuously written text depends not only on the linguistic properties of the words, but also on the quality of these properties learned by the specific reader. The present study was the first attempt to jointly estimate the effects of word properties and individual skills on eye movements during reading among typically developing G3 and G5 children using LMMs. Our results replicated the earlier findings that word properties significantly affect readers’ oculomotor activities during reading. Importantly, we found that word segmenting predicted the G3 children’s FFD and SFD and the G5 children’s FL. We also found that RAN predicted FFD and SFD for the G5 children. Below we discuss how the eye-tracking technique can provide a window to unveil literacy development and how our results contribute to understanding individual differences in reading. This discussion is followed by implications, limitations and the conclusion of the present study.

### Eye-tracking technique and literacy development

Lab-based cognitive methodologies such as reaction time, electroencephalogram (EEG) and functional magnetic resonance imaging (fMRI) often adopt single-word reading paradigms. Psychometric tests used widely in developmental psychology either focus on knowledge about single words, such as morphological and phonological awareness, or measure a global processing speed and ignore how individual units are processed, such as RAN. These methods have limitations in a number of aspects. First, although single-word knowledge and recognition are important aspects of reading development and language cognition, cognitive processes underlying natural reading can be far more complex. In particular, words rarely appear in isolation in everyday life, rather, they appear in sentences and passages. It has been well-documented that during natural reading of continuously written text, readers are expected constantly to generate predications about upcoming words and estimate word centers for saccade-targeting. In addition, readers often process more than one word during each fixation and lexical processing starts before a word is fixated on. These parafoveal words are also attended to for cognitive and linguistic processes of visual forms and phonological information. Second, some psychometric tests, such as word construction and production tasks, measure only readers’ response accuracy but not processing speed, which by itself can provide valuable information. Third, some other psychometric tests, such as RAN, measure global speed but not individual items. However, readers’ processing of individual items can critically reveal the cognitive mechanisms that are involved. Finally, many tasks, such as naming, lexical decision and production, which require explicit and conscious responses to the stimuli, differ from automatic lexical activation during natural sentence reading comprehension.

In contrast, the eye-tracking technology provides a nonintrusive and implicit measurement of cognitive processes during reading at high temporal and spatial resolutions. Existing literature has shown that oculomotor indices, such as fixation location and duration, are influenced by a wide range of variables and are sensitive to reflect cognitive processes underlying reading^11,48^. A natural sentence reading task may arguably offer a more ecologically valid test of reading ability than many other tasks. Indeed, several recent studies endeavored to establish eye-movement changes in reading during literacy development. For instance, Kim et al. provided a first large-scale longitudinal investigation to describe the developmental progression of eye movements during English oral and silent reading between G1 and G3, a time when reading skills develop rapidly^28^. Focusing on German children, Meixner et al. analyzed longitudinal data from G1 to G6 and reported that perceptual span increased rapidly in development from G1 to G2 and remained stable in later grades^77^. Concerning Chinese reading development, a cross-sectional study documented that, although Chinese children’s lexical processing was well-established at G5, they showed a late maturation of saccade generation, arguably attributable to the lack of inter-word spaces and its consequence of online word segmenting^33^.

### Individual differences in reading

Previous research on eye movements during reading focused mainly on how word properties affect oculomotor activities. The current study also replicated these findings that frequency and visual complexity of words strongly affected fixation duration and location. Words that were of higher frequencies and visually less complex were fixated on for shorter time periods and the readers landed further into these words than the less frequent and visually more complex ones. In general, such patterns were consistent across different developmental stages.

As proposed by the Verbal Efficiency Theory^5,6^, the participants’ individual reading skills also affected their word recognition. Previous studies have demonstrated the influence of individual reading skills on eye movements across different orthographies: Fixation duration decreases as age increases, and dyslexic readers exhibit longer fixation durations than typical readers^22,28,30,37^. Kupermann and Van Dyke systematically tested the influence of individual reading skills on eye movements during reading among English readers. They found that RAN and word identification were significant predictors of a number of eye-movement indices across early and late processing stages^15^.

In the present study, we tested four reading skills in Chinese. Of these, RAN and word segmenting were shown to be significant predictors of children’s oculomotor activities during reading. RAN has been demonstrated to be a predictor of reading ability across different writing systems varying in orthographic depth. According to Norton and Wolf, RAN acted as a microcosm of different processes involved in reading^47^. In alphabetic scripts, RAN is a significant predictor of reading speed^3^. In Chinese, RAN has also been a concurrent and longitudinal predictor of reading accuracy and fluency^62,78,79^. RAN has been a language-universal and reliable predictor of reading, possibly because it shares many critical cognitive components with natural sentence reading, such as lexical access of foveal and parafoveal words, phonological decoding and saccade-target selection. In the present study, we found that RAN mainly predicted the G5 children’s reading times. However, it is worth noting that RAN did not predict the children’s FL in either grade. Possibly, individual items are separated by spaces in RAN, which differs from the unspaced layout in Chinese sentences. As such, RAN does not reflect the critical process of word segmenting during Chinese sentence reading.

Besides RAN as a language-universal predictor, individual differences in word segmenting stand out. Chinese differs from alphabetic languages in several different noticeable aspects, with the most salient and unique property of the lack of inter-word spaces. Nevertheless, word-based saccades have still been observed, especially when words are frequent, visually simple and more predictable. Therefore, readers need to segment the words parafoveally before selecting a target position for saccade generation^55^. Chinese readers’ reliance on word boundary segmenting is also supported by experimental evidence of enhanced reading performance and oculomotor activities when explicit word boundary knowledge was provided using alternating text colors while retaining the unspaced layout^57,61,80^. However, interestingly, Pan and colleagues found that G3 children with lower reading abilities benefited less from such an explicit boundary cue^61^. This is because G3 is considered a transition period in which children proceed from learning to read (word decoding) to reading to learn^68^. Children who are still struggling in transitioning from character reading to word reading are considered to be less reliant on word boundary knowledge. Although it takes time to acquire word-segmenting ability during literacy development, it helps children to identify where a word is and facilitates their lexical access of the words. At G5, children are known to have mastered all the basic skills of reading, including word segmenting, and focus on high-level semantic analysis and reading comprehension. Thus, higher word segmenting scores likely reflected a better lexical quality in word representation among the G5 children, making it easier for them to achieve parafoveal word segmenting during sentence reading. Indeed, their gazes landed further into the words as compared to their lower-scored counterparts, suggesting that such word segmenting is important in Chinese reading. The current results also echo previous findings from Chinese adults, who have shown a strong preference of word-based processing^81,82^ and word-based saccade generation^55^.

Our findings have practical implications for teaching and learning Chinese, for typically developing children and for second-language learners. When teaching Chinese, after readers have a relatively good lexical representation, it is important for teachers to stress the unit of words over single characters, which may facilitate their reading acquisition. As a limitation, the current study utilized a cross-sectional design. It is ideal to consolidate the key findings using a longitudinal design to control for cross-individual differences, preferably with a larger sample of participants. Future studies could look further into how other reading skills affect eye-movement characteristics during reading. In conclusion, as the first attempt to simultaneously estimate the effects of linguistic properties of words and individual reading skills on lexical processing during natural sentence reading, language-universal and specific influences were observed at different developmental stages.

In conclusion, the present study illustrated the joint effects of word properties and individual reading skills on eye movement characteristics of Chinese children at grades 3 and 5. Word frequency and number of strokes manifested significant influences on both grades. However, different individual skills exhibited different influences on eye movements at different grades, suggesting that children at different stages may utilize different skills for efficient reading.

## Data Availability

The data and script for analysis are available from the corresponding author on reasonable request.
